# Transparietal Gastric Drainage of Walled-Off Pancreatic Necrosis Using an Endoscopic Technique With the Hot Axios Stent: A Case Report

**DOI:** 10.7759/cureus.86650

**Published:** 2025-06-24

**Authors:** Silvia Fernanda Anaya Meza, Andrés David De León Murillo, Alejandro Alfonso Bedoya Rinaldi, David Raul Cerra Ortegon, Tatiana Paola Pérez García, Pedro Antonio Plaza Ricardo

**Affiliations:** 1 General Practice, Universidad Libre, Barranquilla, COL; 2 General Surgery, Universidad Libre, Barranquilla, COL

**Keywords:** endoscopic, hot axios, lumen-apposing metal stents (lams), necrotising pancreatitis, pancreatitis, stents, ultrasonography

## Abstract

We present the case of a 66-year-old female patient who was hospitalized six months earlier for acute biliary pancreatitis. She presented to the emergency department with a 15-day history of symptoms, which included fever, abdominal pain, and a palpable mass in the mesogastric region. An abdominal computed tomography (CT) scan revealed a peripancreatic fluid collection involving the pancreatic body and head, suggestive of a complication related to the prior episode of acute pancreatitis.

The patient was referred to the gastroenterology service, where a decision was made to pursue an uncommon therapeutic approach. Endoscopic ultrasound (EUS)-guided pancreatic drainage was performed using a lumen-apposing metal stent (LAMS) (Hot Axios, Boston Scientific, Marlborough, MA), followed by scheduled outpatient necrosectomies. A total of three necrosectomies were carried out through the stent, which was subsequently removed one month after its placement. The patient demonstrated a favorable clinical course, with resolution of symptoms and no reported complications.

## Introduction

Acute pancreatitis is an inflammatory process of the pancreas that can involve both local tissues and other organs [1]. It is the third most common gastrointestinal diagnosis in the United States, with annual costs reaching up to 2.6 billion dollars, and its incidence is rising. Necrotizing pancreatitis develops in 5% to 10% of patients with acute pancreatitis and is associated with significant morbidity and prolonged hospital stays. Necrotizing pancreatitis is also linked to high mortality rates, ranging from 11% in cases of sterile necrosis to 32% in infected necrosis and up to 43% in patients with infected necrosis and organ failure [2].

Pancreatic fluid collections may occur as a complication of acute pancreatitis and can sometimes resolve spontaneously. However, a subset of patients with necrotizing pancreatitis may develop well-defined symptomatic collections, classified as walled-off necrosis (WON) [2]. This pathological entity can be managed surgically, percutaneously, or endoscopically. Over the past decade, endoscopic techniques have advanced significantly [1].

Endoscopic treatment of WON has traditionally been performed using plastic stents (PS). Lumen-apposing metal stents (LAMS) have been increasingly used to treat symptomatic WON, as they allow for more effective drainage of necrotic material and enable direct endoscopic necrosectomy through the LAMS due to their larger diameter compared to PS [2]. Moreover, the use of LAMS has been associated with a reduction in the number of required procedures, although they are more expensive devices [3-4]. While some studies favor the use of LAMS, no statistically significant differences have been found in terms of successful drainage or adverse events. Nonetheless, one of the advantages of LAMS is the possibility of performing direct endoscopic necrosectomy [3].

The use of these devices has also been demonstrated in the management of pancreatic collections in the pediatric population [5]. Adverse event rates range from 0% to 50%, with bleeding being the most frequently reported complication [2].

This case report presents a 66-year-old female patient with a medical history of arterial hypertension, type 2 diabetes mellitus, and a prior episode of acute biliary pancreatitis six months earlier. She presented to the emergency department with a 15-day history of abdominal pain and fever. Further diagnostic workup revealed a walled-off pancreatic necrosis as a complication of the previous pancreatitis episode. The patient underwent endoscopic management of the complication with weekly interventions, achieving resolution of the condition within one month.

This case highlights the significance of a novel and innovative approach, as the patient underwent ambulatory endoscopic necrosectomies. The purpose of presenting this case is educational, aiming to share a novel therapeutic approach for the management of patients with pancreatic necrosis, particularly emphasizing the feasibility of performing outpatient endoscopic necrosectomies in clinically stable patients.

## Case presentation

We present the case of a 66-year-old female patient with a medical history of arterial hypertension, type 2 diabetes mellitus, and a hospitalization six months earlier for acute biliary pancreatitis. The patient presented to the emergency department with a 15-day clinical course characterized by progressively worsening upper abdominal pain, rated 6/10 on the visual analog scale, accompanied by documented fever spikes.

On physical examination, vital signs were within normal limits; however, abdominal palpation revealed a palpable mass in the mesogastric region.

Among the admission laboratory tests, the following results were found: hemoglobin: 14 g/dL; hematocrit: 42%; white blood cell count (WBC): 7,400/mm³; platelet count: 235,000/µL; C-reactive protein (CRP): 0.5 mg/L; amylase: 56 U/L; lipase: 70 U/L; serum creatinine: 0.78 mg/dL; aspartate aminotransferase (AST): 29 U/L; alanine aminotransferase (ALT): 49 U/L; total bilirubin: 1.2 mg/dL.

**Figure 1:**
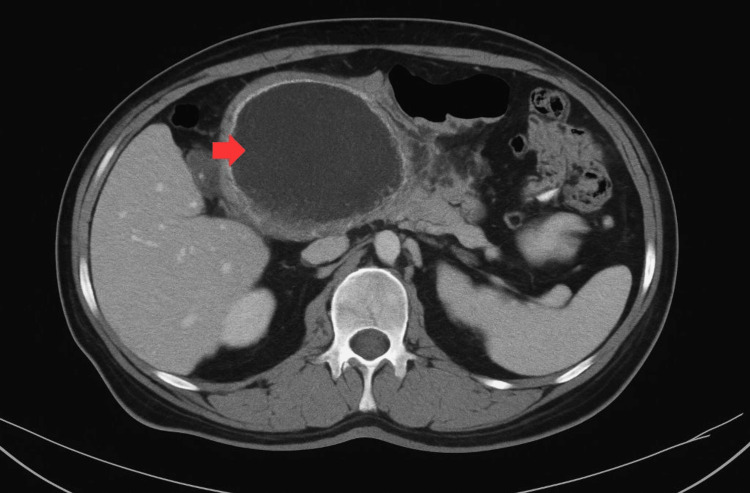
Contrast-enhanced abdominal CT scan showing evidence of a pancreatic collection (red arrow).

Given the patient's medical history, a contrast-enhanced computed tomography (CT) scan of the abdomen was requested (Figure 1), which revealed a well-circumscribed collection located in the head and body of the pancreas, causing compression and displacement of the posterior gastric wall.

A referral to the gastroenterology service was requested, and endoscopic management was chosen using a Hot Axios stent (Boston Scientific, Marlborough, MA), considering the collection size, the predicted need for more than one intervention to resolve the patient's condition, and the higher clinical success rate reported in the literature for LAMS.

**Figure 2:**
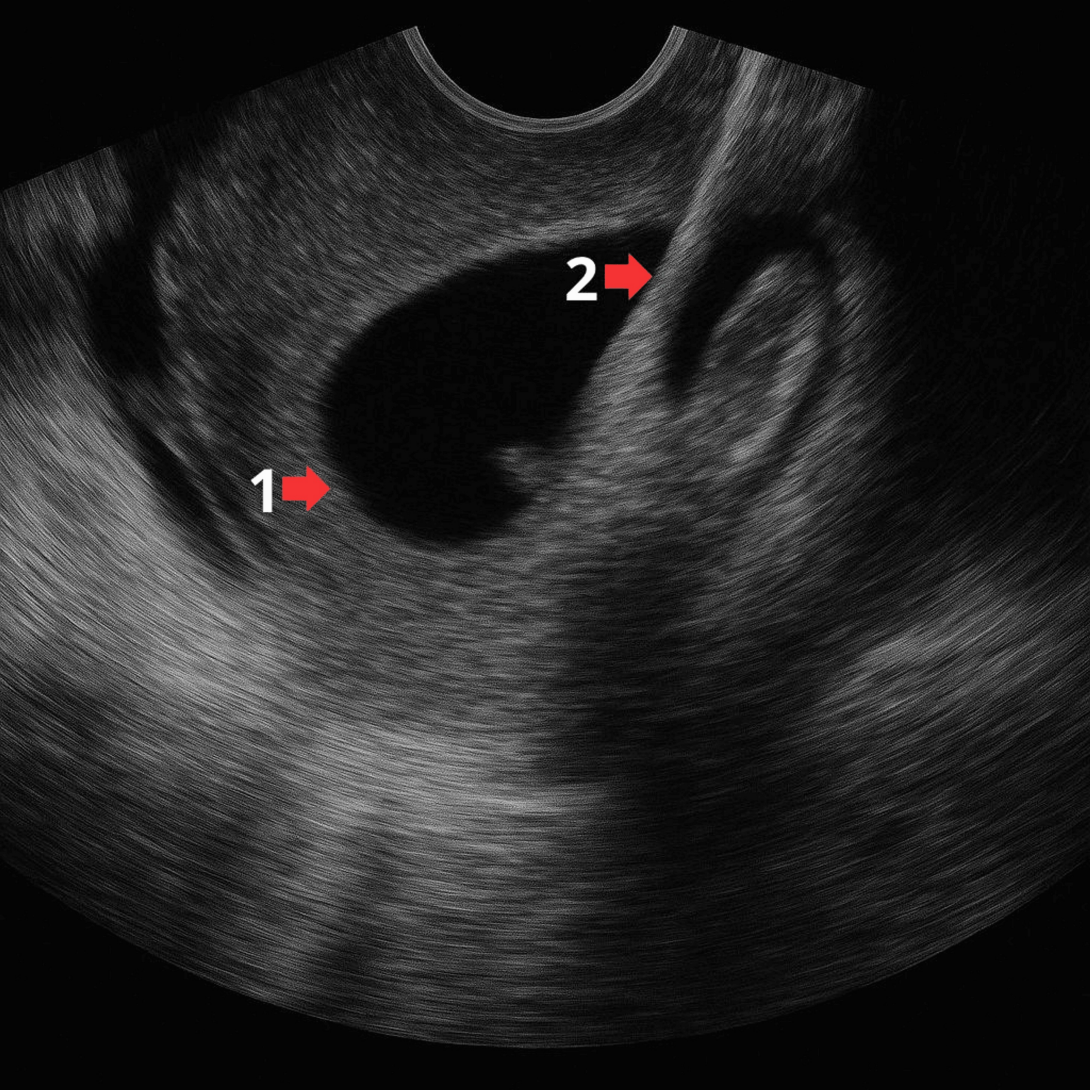
Tuning, deployment, and positioning of the distal cup or bell of the Hot Axios prosthesis in a pancreatic collection (red arrow 1); Pancreatic collection seen by endosonography (red arrow 2)

**Figure 3:**
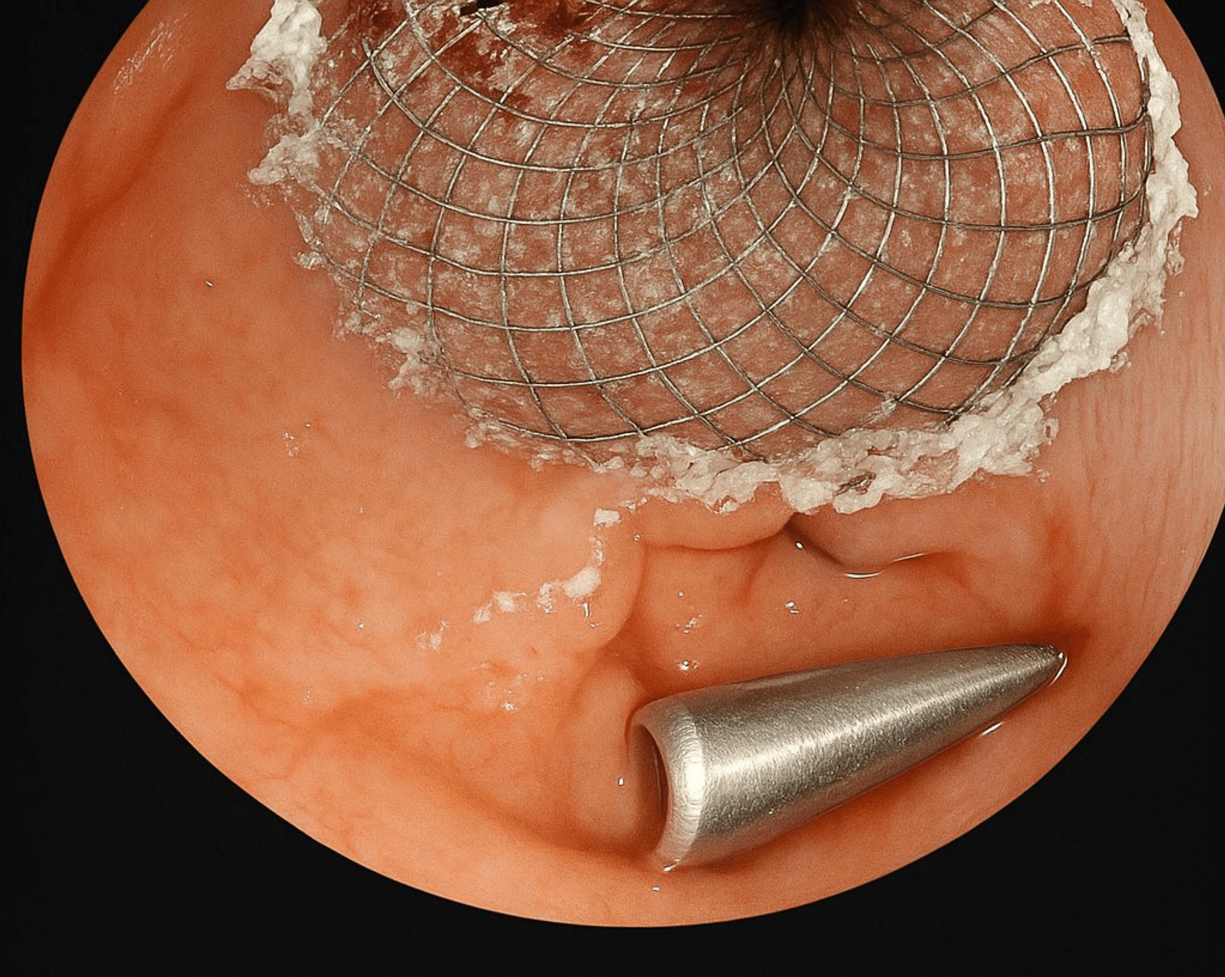
Endoscopic visual confirmation of adequate deployment and positioning of the Hot Axios prosthesis in the stoma placed between the pancreatic necrosis and the gastric chamber.

**Figure 4:**
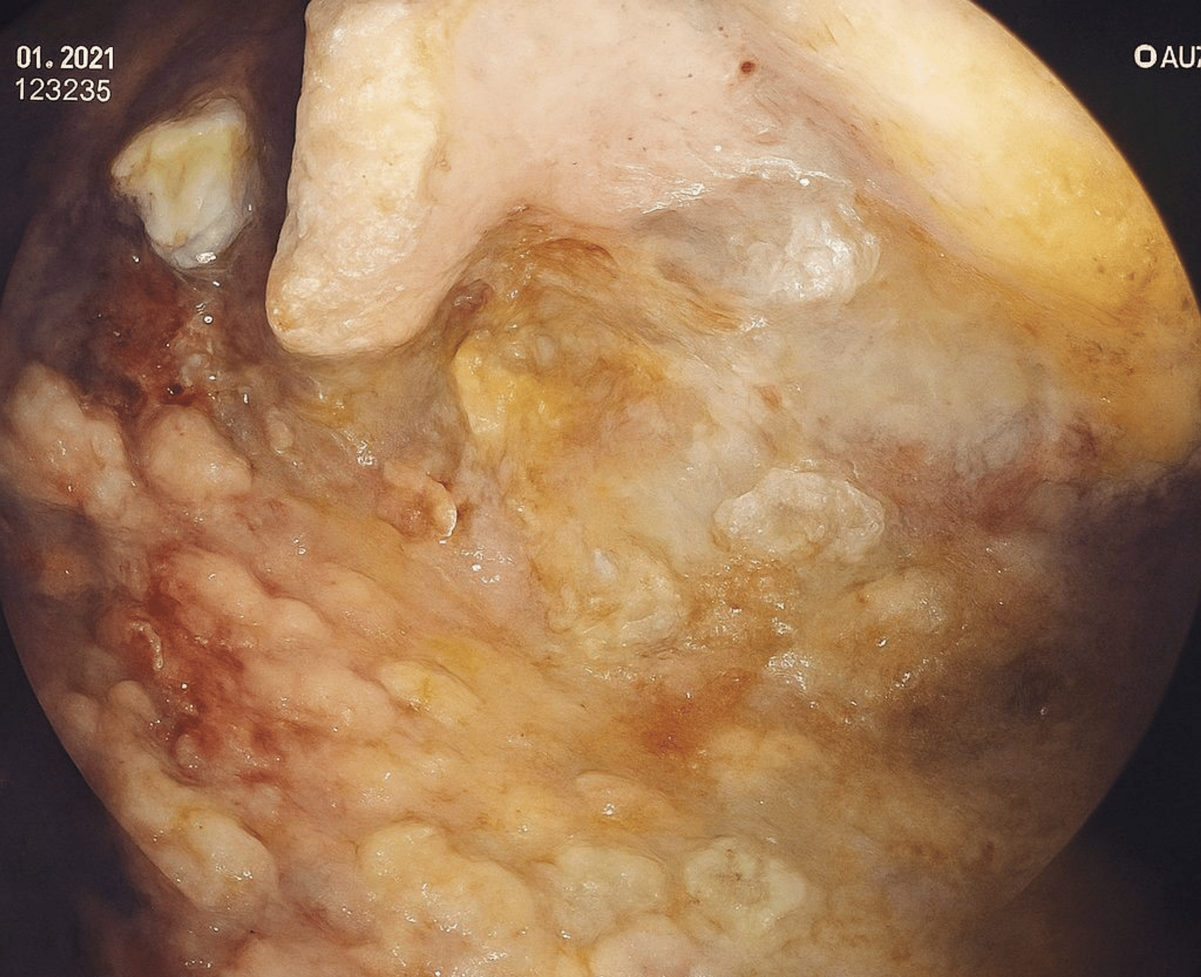
Endoscopic endocavitary view of the pancreatic collection by means of the Hot Axios prosthesis after performing the first necrosectomy session.

**Figure 5:**
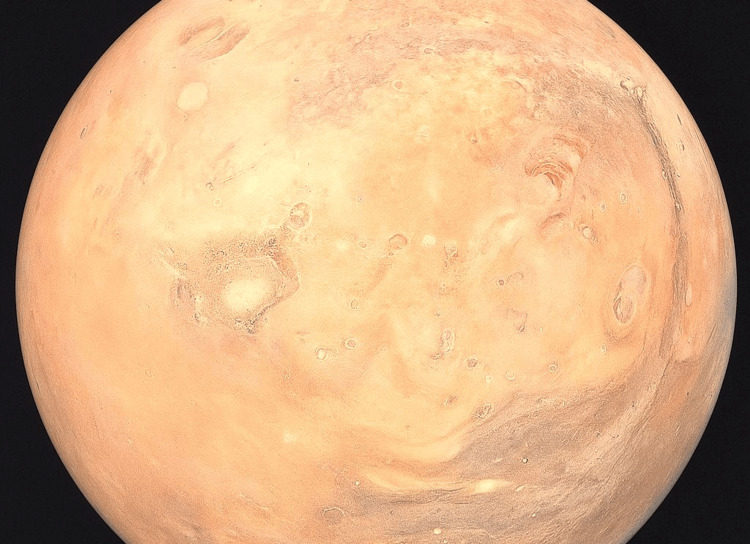
Endoscopic endocavitary view of the pancreatic collection by means of the Hot Axios prosthesis after performing the second necrosectomy session.

**Figure 6:**
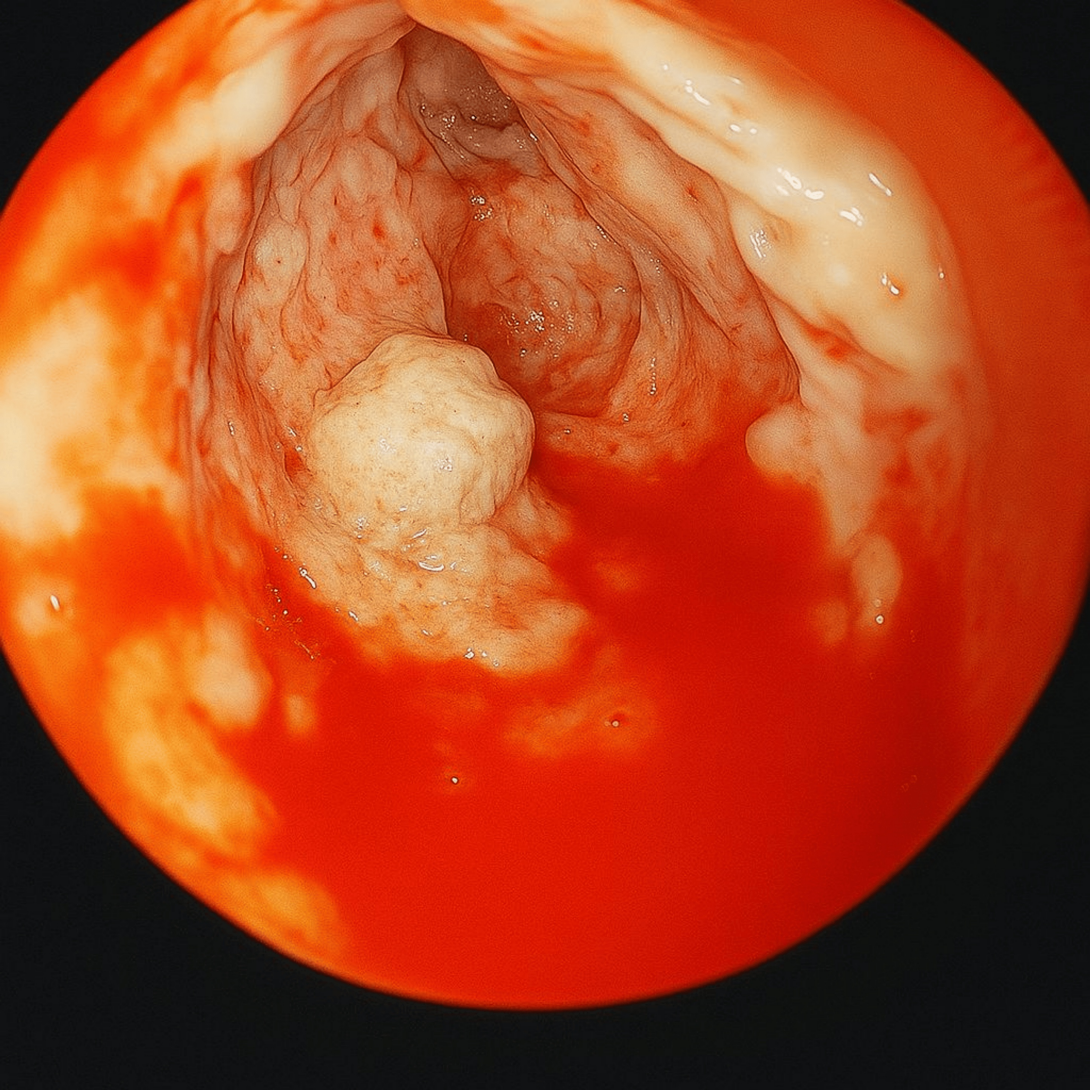
Endoscopic endocavitary view of the pancreatic collection by means of the Hot Axios prosthesis after the third and final necrosectomy session, where adequate granulation tissue is observed.

**Figure 7:**
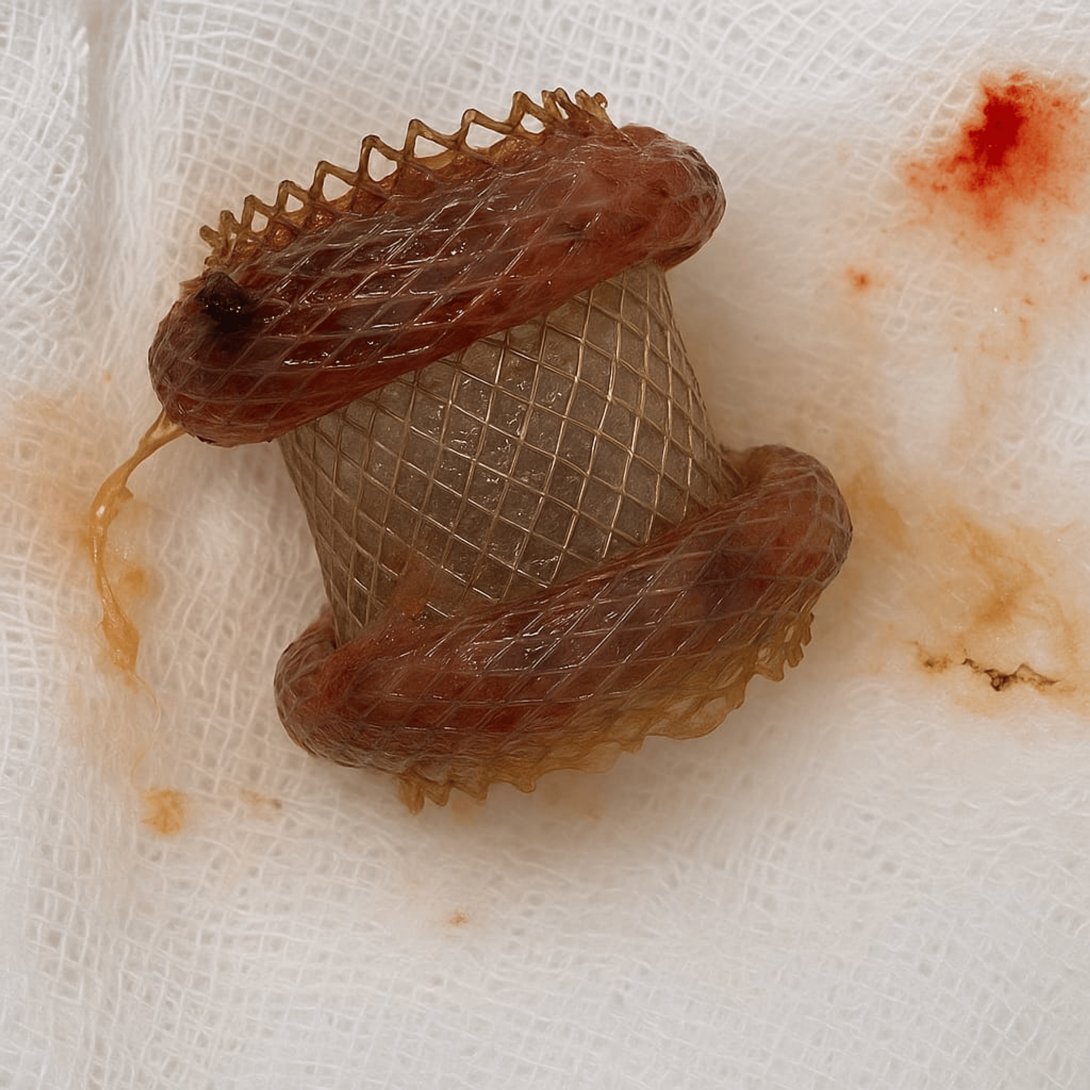
Removal of the Hot Axios stent after the last necrosectomy session.

The patient underwent the first session, during which the deployment of the device was performed under endoscopic ultrasound (EUS) guidance (Figure 2) and finalized once correct positioning was confirmed (Figure 3). The patient was discharged and scheduled for the first necrosectomy one week later (Figure 4). The second necrosectomy was performed one week after the first (Figure 5), and the patient was again scheduled for a third and final necrosectomy one week later (Figure 6). The Hot Axios stent was removed four weeks after its initial placement (Figure 7).

The patient was scheduled for follow-up 15 days after stent removal, showing appropriate clinical progress.

## Discussion

Acute pancreatitis is a common gastrointestinal disorder that can vary from a mild, self-limited condition to a severe and life-threatening illness. In its severe form, pancreatic necrosis and systemic complications can develop, significantly increasing morbidity, prolonging hospitalization, and elevating mortality rates [2]. Among these complications, walled-off pancreatic necrosis represents an encapsulated collection of pancreatic and/or peripancreatic necrosis, which typically develops four weeks after disease onset. The management of WON has undergone significant evolution over recent decades.

Traditionally, open surgical necrosectomy was the cornerstone of treatment for infected pancreatic necrosis. However, it is associated with high complication rates and mortality. As a result, minimally invasive techniques, including image-guided percutaneous drainage, video-assisted retroperitoneal debridement, and endoscopic drainage, have gained prominence, especially following the “step-up” approach described by van Santvoort et al. This strategy promotes an initial conservative and minimally invasive intervention, reserving more aggressive surgical approaches for non-responders [4].

Endoscopic drainage has become one of the preferred methods for managing WON due to its association with reduced systemic inflammatory response, shorter hospital stays, fewer external fistulas, and improved patient quality of life. The technique involves transgastric or transduodenal access to the necrotic collection, followed by the placement of a stent that enables drainage and permits endoscopic necrosectomy in selected cases [2,4].

Two principal types of stents are commonly used for this purpose: double-pigtail plastic stents (DPPS) and LAMS. DPPS were the first to be employed in this setting and are still widely used due to their availability, familiarity among endoscopists, and lower cost. However, their narrow caliber (typically 7 Fr or 10 Fr) limits the drainage of viscous fluid and solid debris, often necessitating multiple procedures or additional interventions such as balloon dilations or catheter placement [1,2].

LAMS were developed to overcome these limitations. These fully covered, large-diameter, biflanged metal stents provide a stable and wide conduit between the gastrointestinal lumen and the necrotic cavity. Their deployment not only facilitates immediate and efficient drainage but also allows direct endoscopic access for necrosectomy without repeated device exchanges. This access permits aggressive debridement when needed, improving overall outcomes [1,3].

Several studies have shown that LAMS are associated with higher clinical success rates (up to 92.63%), faster resolution of necrosis, reduced need for additional interventions, and shorter lengths of stay in both the hospital and intensive care units [1,4]. These findings have contributed to an increasing preference for LAMS in expert centers managing pancreatic necrosis.

Nonetheless, the use of LAMS is not without risks. Recent literature has highlighted a potentially increased risk of bleeding, particularly delayed hemorrhage, which may occur due to the erosion of adjacent vasculature, fistula tract formation, or mechanical trauma during stent removal. These bleeding complications can be severe and occasionally fatal, emphasizing the importance of close monitoring and timing of stent retrieval [2,3]. Moreover, LAMS-related adverse events such as perforation, infection, stent migration, and occlusion occur at rates comparable to those observed with PS, suggesting that neither option is risk-free [2].

Another critical consideration is cost. LAMS are significantly more expensive than DPPS, with reported differences in total procedural costs exceeding $4,000 per case ($20,029 vs. $15,941, respectively) [3]. While the higher initial expense may be offset by decreased length of hospitalization or fewer follow-up procedures, these economic implications must be carefully weighed, especially in resource-limited settings or in institutions where cost-effectiveness is a primary concern.

Despite promising results, the current body of evidence is predominantly based on retrospective studies, case series, and observational data. Randomized controlled trials and prospective, multicenter studies remain scarce, limiting the ability to draw definitive conclusions regarding superiority between the two stent types. Factors such as operator expertise, institutional experience, patient selection, and anatomical variations all play a role in determining outcomes and should be taken into account when choosing the appropriate intervention.

In the present case, the patient presented with clinically stable, symptomatic WON. Due to the absence of systemic inflammatory response, multiorgan failure, or signs of sepsis, a decision was made to perform outpatient endoscopic necrosectomy using LAMS. This approach reflects the growing trend toward ambulatory management in selected patients, with reported safety and efficacy when performed in experienced centers with appropriate follow-up. The patient tolerated the procedure well, and resolution of the collection was achieved with minimal morbidity, underscoring the utility of this technique in carefully chosen scenarios.

## Conclusions

This clinical case highlights the successful use of a minimally invasive endoscopic therapeutic strategy for the treatment of walled-off pancreatic necrosis in a patient with a history of acute biliary pancreatitis. Using the Hot Axios device, sequential drainage and necrosectomies were performed on an outpatient basis, demonstrating a favorable clinical outcome without significant complications. This approach represents a safe and effective alternative to traditional surgical methods, with the added benefit of reducing hospitalization times and improving patient quality of life. Furthermore, it highlights the advancement and practical application of modern endoscopic techniques in the management of severe complications of pancreatitis, contributing evidence of the use of these techniques on an outpatient basis in certain patients.
